# Flaxseed oil and probiotics protect against induced nonalcoholic fatty liver disease (NAFLD) in male rats

**DOI:** 10.1515/biol-2025-1255

**Published:** 2026-02-18

**Authors:** Haddad A. El Rabey, Maha J. Balgoon, Mohammed A. Alsieni, Adel I. Alalawy, Moataz Y. Soliman, Mohamed M. El Hoseeny, Samar M. Rezk, Nadia Bakry

**Affiliations:** Department of Biochemistry, Faculty of Science, University of Tabuk, 71421, Tabuk, Saudi Arabia; Biochemistry Department, Faculty of Science, King Abdulaziz University, Jeddah, Saudi Arabia; Pharmacology Department, Faculty of Medicine, King Abdulaziz University, Jeddah, Saudi Arabia; Hepatology, Gastroenterology and Infectious Diseases Department, Faculty of Medicine, Al-Azhar University, Cairo, Egypt; Internal Medicine Department, Faculty of Medicine, Suez University, Suez, Egypt; Nutrition Department, Mahalla Hepatology Teaching Hospital, GOTHI, Gharbyia, Ministry of Health and Population of Egypt, Cairo, Egypt; Bone Marrow Transplantation and Cord Blood Unit, Mansoura University Children Hospital, Mansoura, Egypt

**Keywords:** NAFLD, omega 3, probiotics, TNF-α, TGF-β, flaxseed oil

## Abstract

This study aimed to evaluate the effect of probiotics and flaxseed oil on nonalcoholic fatty liver disease (NAFLD). 50 male albino rats were divided into five groups (10/each), the 1st negative control group fed on the basal diet (G1) and four other groups subjected to induced NAFLD and then classified into four groups; the 2nd positive control group induced-Non Alcoholic fatty liver disease (G2), the 3rd group was treated with flaxseed oil (G3), the 4th group was treated with live probiotics (G4) and the 5th group was treated with mixed of flaxseed oil and live probiotics (G5). The study was conducted for 8 weeks. GC analysis indicated that the main constituent of flaxseed oil is the omega-3 fatty acid α-linolenic acid methyl ester (44.89 %). The positive control group showed severely altered lipid profile, liver enzymes, transforming growth factor-β (TGF-β), interleukin-6 (IL-6), tumor necrosis factor-α (TNF-α), and liver histology, whereas treating the NAFLD with flaxseed oil or probiotic strains or their mix significantly improved lipid profile, liver enzyme, transforming growth factor-β (TGF-β), inflammatory markers (tumor necrosis factor-α (TNF-α) and interleukin-6 “IL-6”), and significantly decreased oxidative stress levels than the positive control group. Flaxseed oil and probiotics, separately and in combination, confer hepatoprotective effects against induced NAFLD in rats.

## 1 Introduction

Recently, the etiology of chronic hepatic diseases has changed due to the decrease of viral hepatitis and the growing new epidemic of a wide range of metabolic complaints like steatosis, nonalcoholic fatty liver disease (NAFLD) and nonalcoholic steatohepatitis (NASH) [1], 2]. Nonalcoholic fatty liver disease (NAFLD), recently defined as metabolic-associated fatty liver disease (MAFLD), when fat accumulates in hepatic tissues due to evident causes such as viral hepatitis, alcohol use, or medications [3], 4]. NAFLD has become a rapidly growing major health issue in developed countries affecting nearly 30 % of the general global adult population [5].

The first stage of NAFLD is simple hepatic steatosis (excessive fat loading in the hepatocytes), followed by the evolution of steatosis into cirrhosis of the liver, caused by inflammation and fibrosis, causing irreparable liver damage [6]. NAFLD is the most common chronic liver disease in adults and children [7]. In NAFLD patients, simple hepatic steatosis can progress to steatohepatitis, fibrosis, and cirrhosis (Brown, Kleiner 2016). On the other hand, NAFLD is more common among overweight and obese adults [8]. NAFLD could be a risk factor for the incidence of another metabolic syndrome, not only one [9]. NAFLD is supposed to affect nearly one-third of people worldwide, especially in developed countries [10].

More than a hundred trillion microbes are housed in the gastrointestinal tract (GIT), termed gut microbiota that promote innate and adaptive immunity by communicating and interacting with each other to maintain healthy immune activity via the influence on the immune system through a complex network of pathways that maintain the balance of immune tolerance and immune capacity [11]. In addition, bacterial interactions of probiotics can stimulate intestinal immune cells and commensal microorganisms to modulate specific immune functions, stabilize immunity, promote health, and consequently provide a health benefit to the host [12]. Animal studies have shown that modifying the gut microbiota with probiotic preparations can help with NAFLD [13].

On the other hand, human data on the involvement of gut microbiota in NAFLD and its therapeutic usage is still in the early stages [14]. Probiotics may prevent spontaneous ascorbate oxidation, reducing activity, chelating metal ions, free radicals scavenging such as superoxide anion, hydrogen peroxide and reactive oxygen species that are constantly produced in the human body, and preventing lipid peroxidation [15]. Moreover, probiotics also decrease serum cholesterol in NAFLD patients [16]. On the other hand, probiotics regulate liver inflammation by balancing the synthesis of anti- and pro-inflammatory cytokines tumor necrosis factor-α (TNF-α), improving liver function, and reducing the content of hepatic fatty acids and their β-oxidation [17].

Omega-3 fatty acids must be received in the diet or as supplementation because they are essential fatty acids [18], [19], [20]. They aid in the determination of inflammation and change the function of vascular and carcinogen biomarkers, thus reducing the coronary heart disease (CHD) risk (CVD) and cancer. Omega-3 fatty acids offer considerable protection in contradiction to other chronic diseases such as diabetes mellitus (DM) (metabolic disease), osteoporosis, obesity, and bone fractures [21]. Importantly, the outcome after long-term pharmacological therapy in NAFLD patients is not well investigated. Physicians have turned their attention to other therapeutic choices, such as omega-3 fatty acids and probiotics, due to patient non-compliance with lifestyle changes and different results with other treatments [14], 21]. Also, the omega-3 fatty acids-rich diet decreased insulin resistance, intra-hepatic triglyceride deposition, and improved steatohepatitis in NAFLD rats [22], [23], [24]. Omega-3 fatty acids supplements were associated with lowering lipid peroxidation indicated by lower plasma malondialdehyde (MDA) in humans and animals [25].

This study was focused on evaluating the effect of omega-3 fatty acids (in the flaxseed oil) and probiotics in protecting against induced NAFLD in male rats.

## 2 Materials and methods

### 2.1 Materials and chemical

Flaxseed oil was purchased from the local market; the 14 alive pure probiotic strains (*Bifdobacterium bifdum*, *Bifdobacterium longum*, *Bifdobacterium breve*, *Lactobacillus rhamnosus*, *Bifdobacterium lactis*, *Lactobacillus gasseri*, *Lactobacillus fermentum*, *Lactobacillus bulgaricus*, *Lactobacillus plantarum*, *Lactobacillus paracasei*, *Lactobacillus acidophilus*, *Lactobacillus casei*, *Lactobacillus reuteri* and *Lactobacillus salivarius*) were purchased from Puritan’s Pride, NC company (USA).

### 2.2 GC analysis

The flaxseed oil used in treating the experimental animals was analyzed using the PerkinElmer Autosystem GC (USA). The flaxseed oil was kept in Amber vial at 4 °C and were checked two weeks after they were opened. Using methanolic HCl, lipids (100 µL oil) were turned into fatty acid methyl esters (FAMEs) through direct transesterification as follows: The glass tube was sealed and filled with 100 µL of oil and 2 mL of 3 *N* methanolic HCl. It was then heated for 60 min at 80 °C. After it has cooled, add 2 mL of distilled water and 2 mL of hexane. Vortex and spin at 2,000×*g* for 5 min. The top layer of hexane with FAMEs was kept, and an internal standard (heptadecanoic acid methyl ester, C17:0, 50 μg/mL) was added. Anhydrous Na2SO4 was used to dry the extracts, which were then filtered, evaporated under *N*2, and put back together in 500 µL of hexane for GC analysis. We used a gas chromatograph with a flame ionization detector (GC–FID) to do the GC analysis. The instrument needed a column DB-23 (60 m × 0.25 mm ID × 0.25 µm film) (or an equivalent polar capillary column for FAMEs), a carrier gas of helium at 1.0 mL/min, an injection volume of 1 µL, a split ratio of 50:1, an injector temperature of 250 °C, and a detector temperature of 260 °C. Set the oven to 100 °C for 2 min, then raise the temperature to 175 °C at a rate of 10 °C/min for 10 min, and finally raise the temperature to 230 °C at a rate of 4 °C/min for 6 min. We found FAMEs by looking at how long they stayed in the system compared to a 37-component FAME standard mix (Supelco) and measuring their peak area against the internal standard. The results are shown as a percentage of the total number of fatty acids that were found (area %). We did the tests on all of the samples twice and reported the average value. We used calibration standards (serial dilutions of FAME mix) to make sure that the limit of detection and linearity were right for the main FAMEs.

### 2.3 Animals and diet

Fifty adult male rats (Sprague-Dawley strain) weighing 180 ± 5 g were obtained from the Egyptian Agricultural Research Center and housed at 23 ± 2 °C. The rats were randomly divided 10/stainless steel cages and kept under surveillance for one week before the experiment and fed a basal diet and water *ad libitum*.

### 2.4 Experimental design

All biological experiments are carried out according to the Institute of Laboratory Animal Resources rules, Commission on Life Sciences, National Research Council in 1996. Investigations were performed at Mansoura University, Mansoura, Egypt. The rats were subjected daily to a physical examination to observe their health condition. The animals were housed in stainless cages with food and water *ad libitum* at 23 ± 2 °C room temperature, 60–70 % relative humidity, and the light-dark cycle of 12 h–12 h, during the experiment. The rats were divided into five groups (*n* = 10), a negative control group (G1, received a daily dose of 1 mL of 0.9 % saline solution) and four groups with induced NAFLD: untreated positive control (G2), flaxseed oil (G3, received 1.7 ml/kg flaxseed oil, orally according to Wani et al. [26], live probiotics (G4, 140 mg/kg powder in the diet “the colony forming unit (CFU) is 107/g”, according to Wong et al. [27], and flaxseed oil + probiotics (G5, with the same concentration of G3 and G4). The NAFLD was induced in the four groups by administration of a high-fructose corn syrup (55 % fructose and 45 % glucose) mixed with drinking water for two weeks [28]. The treatment was diluted in 1 mL of 0.9 % saline solution and were administered daily by gavage for 8 weeks. Rats were fed standard rodents’ basal diet during the 8 weeks experiment time.

**Ethical approval:** The research related to animal use has been complied with all the relevant national regulations and institutional policies for the care and use of animals, and has been approved by the IRB Mansoura Faculty of Medicine, Mansoura University (code number: R.19.10.659).

### 2.5 Dissection, blood collection and biochemical analyses

#### 2.5.1 Sample collection

At the end of the experiment, the rats were euthanized using carbon dioxide in their cages (by displacing about 30–70 % of the cage volume by carbon dioxide flow per minute), which caused narcosis. Then cervical dislocation was achieved and dissected to obtain the liver. Blood samples were collected into tubes with or without anticoagulants. Rat blood samples were centrifuged at 2,000 g for 10 min at 4 °C, then aliquoted for the various analytical tests and stored frozen at – 20 °C until analysis.

#### 2.5.2 Determination of biochemical parameters

Measurements were made using automated and standardized methods, serum alanine aminotransferase (ALT), aspartate aminotransferase (AST) enzymes, total protein (TP), albumin, total bilirubin and alkaline phosphatase (ALP) were estimated by a commercial kit Human according to the instructions of the supplier.

#### 2.5.3 Antioxidant concentration in serum

The serum concentration of catalase (CAT), superoxide dismutase (SOD), total antioxidant capacity (TAC), and the lipid peroxidation marker; malondialdehyde (MDA) were measured according to the methods described by Chance and Mackley [29], DeChatelet al. [30], Beutler et al. [31], and Stocks and Dormandy [32], respectively. Glucose levels were measured using commercial assay kits (Human company). Lipid profiles [triglycerides (TG), total cholesterol (TC), high-density lipoprotein (HDL), low-density lipoprotein (LDL)] were calculated by enzymatic colorimetric methods using commercial assay kits (Spinreact kits). TNF-α, IL-6 and transforming growth factor-β (TGF-β) levels were analyzed with enzyme-linked immunosorbent assay (ELISA) using a commercial kit: (TNF-α, L-6: R&D Systems rat kit), (Biovision rat kit), respectively.

### 2.6 Histopathological study

Liver specimens were collected and fixed in 10 % formalin, then dehydrated in increasing ethanol concentrations (70, 80 and 90 %), cleared in xylene and embedded in paraffin. Finally, microtomic sections of about 4–5 μm were prepared, stained with hematoxylin and eosin dye, and then examined and photographed [33].

### 2.7 Statistical analysis

Data were analyzed using SPSS (statistical package for social science) program (SPSS, Inc, Chicago, IL, USA) 21.0 software. Data are presented either as *n* (%) or mean ± SD. One-way analysis of variance (ANOVA) test was followed by a post hoc test (Tukey HSD test). *P* < 0.05 is considered significant. *P*-value < 0.05 was used to determine the significance. For correlations between variables (Pearson’s rank) was used. When *P*-value <0.05 it is accepted as significant.

## 3 Results

### 3.1 GC analysis of flaxseed oil

**Figure 1:**
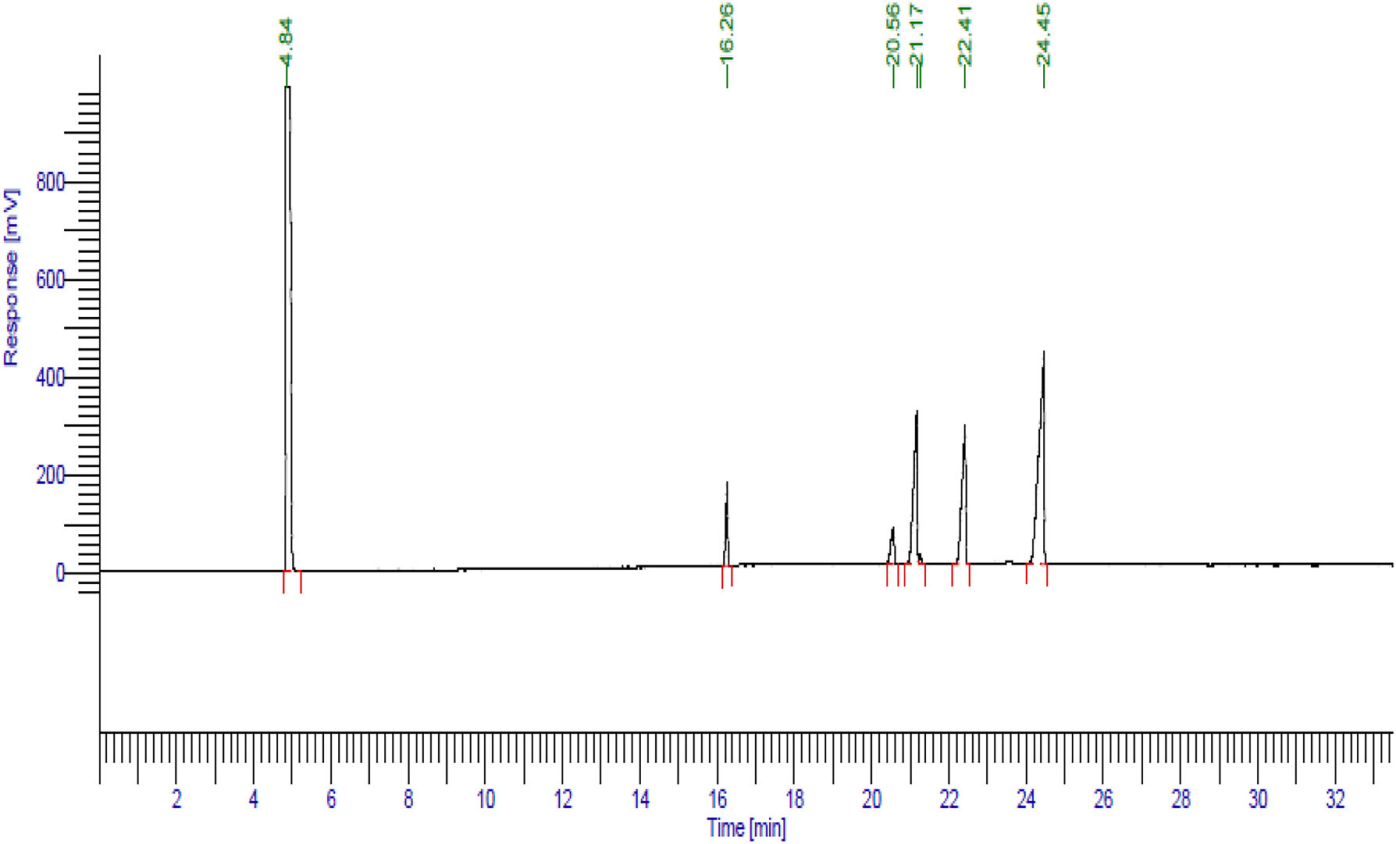
GC analysis of the flaxseed oil showing its bioactive constituents of fatty acids.

The GC analysis of the flaxseed oil showed that the main constituents is 44.89 % Linolenic acid methyl ester (C18:3*n*3) (an omega-3 fatty acid), 24.17 % α-linolenic acid methyl ester (C18:2*n*6c), 22.32 % Oleic acid methyl ester (C18:1*n*9c), 6.42 % Palmitic acid methyl ester (C16:0) and 5.01 % Stearic acid methyl ester (C18:0) (Figure 1).

### 3.2 Liver function

**Figure 2:**
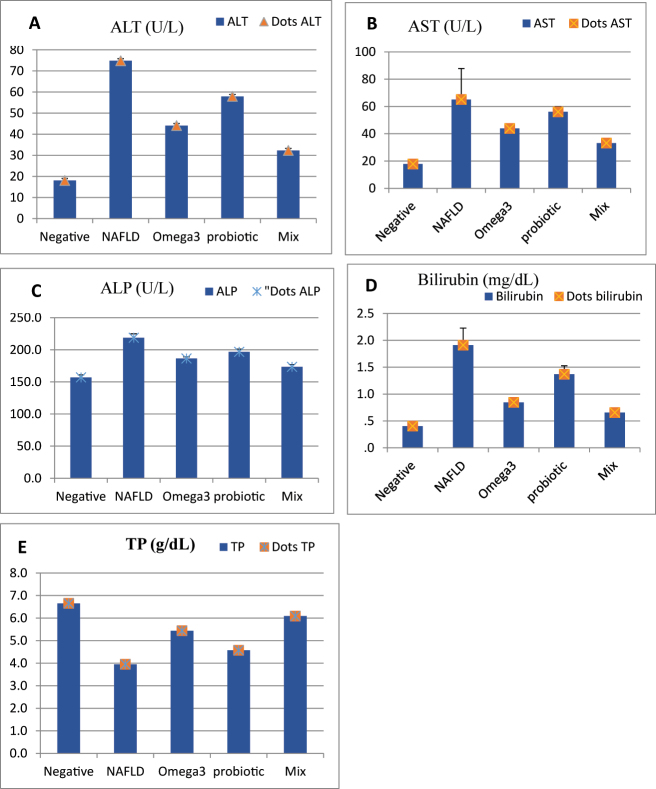
Liver function parameters: A; alanine aminotransferase (ALT), B; aspartate aminotransferase (AST), C; alkaline phosphatase (ALP), D; Bilirubin and E; total protein (TP).

Figure 2 (A, B, C, D, E) and supplementary Table 1 show that serum ALT, AST, ALP and bilirubin significantly (*P* < 0.05) increased in NAFLD-induced rats (G2) compared to negative control G1. In contrast, the levels of TP were decreased. However, these parameters were significantly (*P* < 0.05) improved in all treated groups compared to the NAFLD-induced rats (G2) by treating with flaxseed oil in G3, probiotics in G4 and their mix in G5, especially in G5 that received mix of flaxseed oil and probiotics as compared to other treated rat groups (group 3, 4) (*P* < 0.05).

Table 1 shows that the administration of high-fructose corn syrup in G2 increased the levels of serum glucose, TC, TG and LDL, and decreased the HDL, compared to the negative control (G1). While treating these rats for eight weeks with flaxseed oil, probiotics, and their mix led to a significant reduction of TC and TG and increased HDL. Significant differences (*P* < 0.05) were observed for TG and TC when comparing changes in flaxseed oil and probiotic groups (G3 and G4, respectively), as shown in Table 1. In addition, blood glucose levels were decreased in group 5 compared to other treated groups (G3, G4) and NAFLD-induced G2. Treating with the mix of flaxseed oil and probiotics in G5 was more efficient than using flaxseed oil in G3 or probiotics in G4 in improving these altered parameters.

**Table 1:** Plasma concentrations of serum glucose, total cholesterol TC, TG, HDL, LDL, in control and the different treated groups.

Variables	Glucose (mg/dl) *n* (10)	TC (mg/dl) *n* (10)	TG (mg/dl) *n* (10)	HDL (mg/dl) *n* (10)	LDL (mg/dl) *n* (10)
G1 (negative control)	94.3 ± 9.16	162.4 ± 5.9	140.6 ± 24.5	43.7 ± 3.7	95.0 ± 7.7
G2 (positive NAFLD)	180.7 ± 11.4^a^	276.0 ± 7.7^a^	238.2 ± 22.7^a^	15.9 ± 2.9^a^	211.3 ± 8.4^a^
G3 (flax seed oil)	141.2 ± 2.69^a^#	217.9 ± 11.8^a^#	183.6 ± 9.5^a#^	32.6 ± 1.8^a#^	147.3 ± 10.8^a^#
G4 (probiotics)	154.3 ± 2.75^a^#	254.8 ± 2.8^a^#	216.5 ± 6.6^a#^	24.1 ± 1.7^a#^	186.9 ± 2.3^a^#
G5 (flax seed oil + probiotics)	129.5 ± 1.90^a^#	192.3 ± 3.4^a^#	179.3 ± 5.4^a#^	39.5 ± 1.9^a#^	116.6 ± 3.2^a^#

^a^significant at *P* < 0.05 compared to the negative control group (G1) and # significant at *p* < 0.05 as compared to control NAFLD-induced rats (G2).

### 3.3 Oxidative stress indices and serum antioxidants

**Figure 3:**
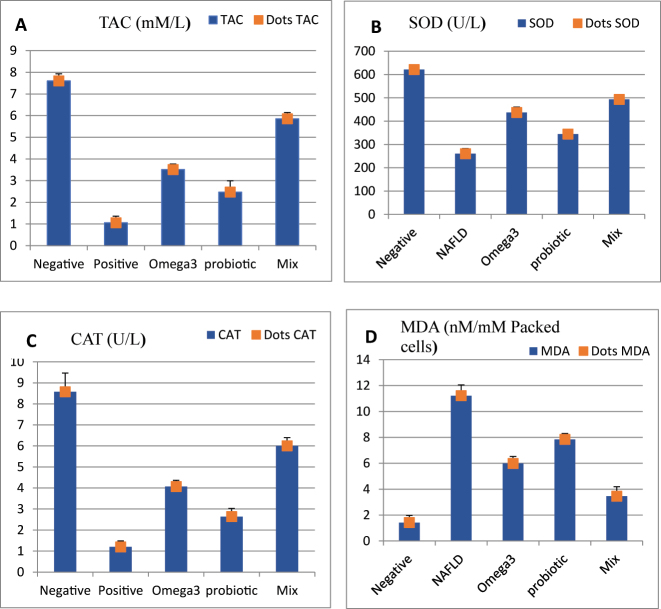
Oxidative stress parameters: A; total antioxidant capacity, B; superoxide dismutase (SOD), C; catalase (CAT) and D; malondialdehyde (MDA).

The oxidative stress markers in the positive control group (G2) showed a significant decrease (P < 0.05) in total antioxidant capacity (TAC), catalase (CAT), and superoxide dismutase SOD levels compared to the negative control group (G1) as shown in Figure 3 (A,B,C,D) and supplementary Table 2. In contrast, MDA levels were significantly (P < 0.05) increased relative to that in the negative control group (G1). TAC, CAT and SOD levels were significantly (P < 0.05) increased in the treated groups (G3, G4 and G5) compared to that of G 2, whereas MDA levels were significantly (P < 0.05) decreased. Flaxseed oil and probiotics mix group (G5) showed more improvement than flaxseed oil or probiotics alone in G3 and G4, respectively.

Table 2 shows that serum IL-6, TGFβ, and TNFα were significantly (P < 0.05) increased in NAFLD-induced rats (G2) compared to that of the negative control group (G1). However, these parameters were significantly (P < 0.05) decreased in all treated groups compared to the NAFLD-induced rats (G2) (P < 0.05).

**Table 2:** IL-6, TGF-β, and TNF-α in the studied groups.

Variables	IL-6 (pg/mL)	TGF-β (pg/mL)	TNF-α (pg/mL)
G1 (negative control)	13.89 ± 0.641	64.15 ± 5.21	17.71 ± 1.72
G2 (positive NAFLD)	37.56 ± 2.65^a^	186.10 ± 11.31^a^	49.09 ± 2.84^a^
G3 (flax seed oil)	21.73 ± 2.50^a#^	128.75 ± 3.69^a#^	29.44 ± 1.99^a#^
G4 (probiotics)	26.56 ± 4.08^a#^	146.34 ± 24.41^a#^	38.19 ± 1.46^a#^
G5 (flax seed oil + probiotics)	15.46 ± 0.53^a#^	88.69 ± 4.79^a#^	21.26 ± 1.08^a#^

^a^significant at *P* < 0.05 compared to the negative control group (G1) and # significant at *p* < 0.05 as compared to control NAFLD-induced rats (G2).

A significant (*P* < 0.05) decrease in these parameters was observed in G5 rats, which received combined therapy of flaxseed oil and probiotics, compared to other treated groups (G3, G4) (*P* < 0.05). Table 3 shows that the diseased groups’ mean value of final rat weight and liver weight were within the obesity range and significantly (*P* < 0.05) higher than the normal control group in G1. A significant difference (*P* < 0.05) could be detected between different groups of rats over the eight weeks. The mean final weight and liver weight of the NAFLD-induced group in the 4th week were higher than the treated group with both flaxseed oil and probiotics, which was better than that of the weight of flaxseed oil-treated rats and better than the weight of the probiotics-treated rats.

**Table 3:** Liver weight, initial weight and final weight in the studied groups.

Variables	Liver weight (g)	Initial weight (g)	Final weight (g)
G1 (negative control)	3.09 ± 0.099	215.4 ± 8.99	256.3 ± 0.45
G2 (positive NAFLD)	5.81 ± 0.213^a^	213.4 ± 11.72	331.0 ± 9.35^a^
G3 (flax seed oil)	4.37 ± 0.235^a^#	211.3 ± 10.656	283.1 ± 4.35^a^#
G4 (probiotics)	5.02 ± 0.225^a^#	215.0 ± 10.57	302.2 ± 8.08^a^#
G5 (flax seed oil + probiotics)	3.71 ± 0.144^a^#	214.6 ± 11.75	274.1 ± 0.2.923^a^#

^a^Significant at *P* < 0.05 compared to the negative control group (G1) and # significant at *p* < 0.05 as compared to control NAFLD-induced rats (G2).

A positive correlation was detected between measured oxidative stress and inflammatory markers. Also, a positive correlation was observed between the investigated antioxidants (SOD, CAT, and TAC) and with each other, and a negative correlation was observed between antioxidant biomarkers and MDA. In addition, a positive correlation have been found between liver injury markers and inflammatory cytokines (Table 4).

**Table 4:** Correlations coefficient^®^ values of the measured parameters in all groups.

	ALT	AST	TP	Albu	Chol	TG	Il-6	TGF-β	TNF-α	MDA	SOD	CAT	TAC
ALT	–	0.856^a^	−0.974^a^	−0.974^a^	0.972^a^	0.899^a^	0.939^a^	0.947^a^	0.964^a^	0.967^a^	−0.969^a^	−0.947^a^	−0.965^a^
AST	0.856^a^	–	−0.873^a^	−0.837^a^	0.840^a^	0.732^a^	0.759^a^	0.829^a^	0.848^a^	0.828^a^	−0.865^a^	−0.852^a^	−0.844^a^
TP	−0.974^a^	−0.873^a^	–	0.988^a^	−0.985^a^	−0.870^a^	−0.922^a^	−0.944^a^	−0.970^a^	−0.967^a^	0.972^a^	0.950^a^	0.962^a^
Albu	−0.974^a^	−0.837^a^	0.988^a^	–	−0.987^a^	−0.899^a^	−0.921^a^	−0.945^a^	−0.960^a^	−0.968^a^	0.973^a^	0.955^a^	0.969^a^
Chol	0.972^a^	0.840^a^	−0.985^a^	−0.987^a^	–	0.888^a^	0.910^a^	0.931^a^	0.953^a^	0.959^a^	−0.967^a^	−0.953^a^	−0.962^a^
TG	0.899^a^	0.732^a^	−0.870^a^	−0.899^a^	0.888^a^	–	0.827^a^	0.821^a^	0.862^a^	0.874^a^	−0.894^a^	−0.880^a^	−0.875^a^
IL-6	0.939^a^	0.759^a^	−0.922^a^	−0.921^a^	0.910^a^	0.827^a^	–	0.941^a^	0.939^a^	0.926^a^	−0.901^a^	−0.875^a^	−0.907^a^
TGF-β	0.947^a^	0.829^a^	−0.944^a^	−0.945^a^	0.931^a^	0.821^a^	0.941^a^	–	0.938^a^	0.951^a^	−0.937^a^	−0.933^a^	−0.953^a^
TNF-α	0.964^a^	0.848^a^	−0.970^a^	−0.960^a^	0.953^a^	0.862^a^	0.939^a^	0.938^a^	–	0.966^a^	−0.947^a^	−0.925^a^	−0.941^a^
MDA	0.967^a^	0.828^a^	−0.967^a^	−0.968^a^	0.959^a^	0.874^a^	0.926^a^	0.951^a^	0.966^a^	–	−0.952^a^	−0.946^a^	−0.958^a^
SOD	−0.969^a^	−0.865^a^	0.972^a^	0.973^a^	−0.967^a^	−0.894^a^	−0.901^a^	−0.937^a^	−0.947^a^	−0.952^a^	–	0.969^a^	0.960^a^
CAT	−0.947^a^	−0.852^a^	0.950^a^	0.955^a^	−0.953^a^	−0.880^a^	−0.875^a^	−0.933^a^	−0.925^a^	−0.946^a^	0.969^a^	–	0.971^a^
TAC	−0.965^a^	−0.844^a^	0.962^a^	0.969^a^	−0.962^a^	−0.875^a^	−0.907^a^	−0.953^a^	−0.941^a^	−0.958^a^	0.960^a^	0.971^a^	–

(^a^) Significant, *p* > 0.05 in each correlations.

### 3.4 Liver histopathology

**Figure 4:**
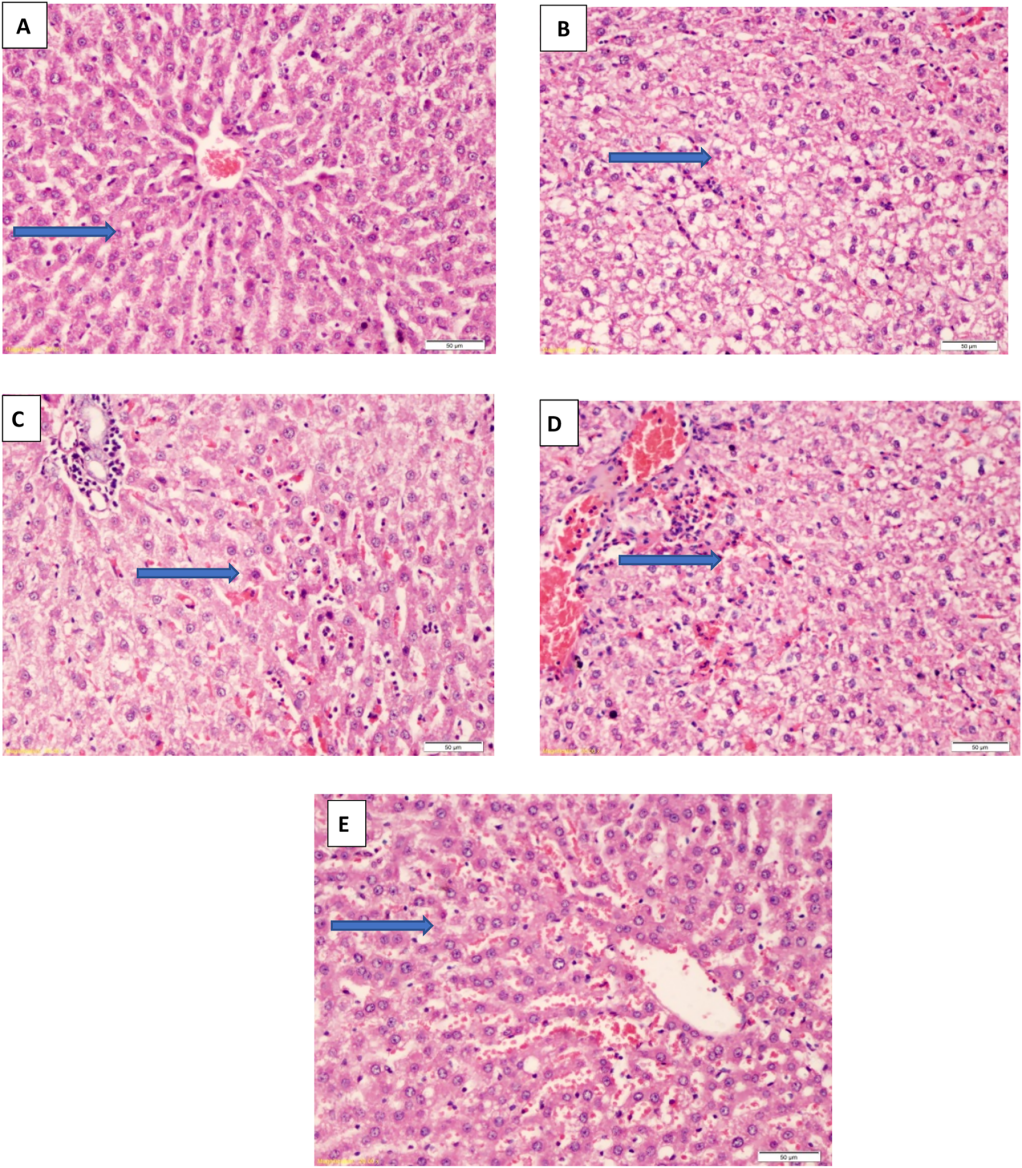
Microscopic images of liver tissue under study; A: G1 group showing the normal histological structure of hepatic lobule, normal sinusoid no inflammation, nor necrosis, liver tissue with preserved normal architecture (arrow), B: G2 (NAFLD-induced) showing vacuolated hepatocyte (fatty changes) with marked sinusoidal inflammation and prominent von – kupffer cells (arrow), C: G3 (treated with flax seed) showing improved regenerated hepatocytes with minimal inflammation (arrow), D: G4 (treated with probiotics) showing improved fatty hepatocyte with mild degeneration, and mild inflammation with less sinusoidal congestion (arrow), E: G5 (treated with flax seed + probiotics) showing nearly normal hepatocyte with very mild inflammation (arrow). (H & E-stained X = 200).

The histological examinations of the liver in the studied groups are shown in Figure 4(A–E). Figure 4A shows the untreated negative control group (G1) with normal hepatic tissues. Severely damaged liver tissues are shown in G2 because of the induced NAFLD (Figure 4B) with congested hepatic vein, fatty infiltrated and degenerated hepatocytes, and bridging necrotic cells with sinusoid degenerated biliary duct. Whereas, Figure 4C shows hepatic tissues of G3 rats treated with flaxseed oil showing regenerated hepatocytes with minimal fatty cells and moderate sinusoidal inflammation with prominent non-kupffer cells. Figure 4D shows hepatic tissues of rats treated with probiotics in G4 with regenerated hepatocyte with mild inflammation. Nearly 5 % of the cells vacuolated with less sinusoidal congestion. In addition, group 5 rats treated with both flaxseed oil and probiotic (Figure 4E), showed hepatic tissues with nearly normal hepatocyte with very mild inflammation of portal area, less interstitial edema and very mild congestion. G5 (treated with a mix of probiotics and flaxseed oil showed nearly normal hepatocytes without any signs of inflammation.

## 4 Discussion

Hitherto, no licensed pharmacological therapies for treating NAFLD have been developed, and promising interventions appear to be effective and an emerging therapeutic landscape of new agents that target MAFLD [3], 4]. NAFLD was induced in the second group (G2) because fructose activates protein fructosylation that promotes the production of reactive oxygen species in rats’ livers [17], 34]. While, natural products rich in phenolic compounds are very important for protecting the liver by reducing oxidative stress and improving the lipid profile [35], [36], [37].

In the current study, flaxseed oil was used in treating the NAFLD due to its richness in bioactive phenolic compounds [23], 24]. The GC analysis of the flaxseed oil revealed that the omega-3 fatty acid, linolenic acid is the main bioactive constituent followed by Linoleic acid methyl ester (C18:2*n*6c), oleic acid methyl ester (C18:1*n*9c), palmitic acid methyl ester (C16:0) and stearic acid methyl ester (C18:0). These bioactive constituents have many health benefit correlated to heart and brain health thanks to their antioxidant activity [23], 24], 38], 39].

Flaxseed oil and probiotics-a promising medicine still under validation-were used to treat NAFLD and their effect on liver functions and fatness after induction of NAFLD in rats via high fructose diet supplementation [28]. Previous research has sought to establish a link between high-fructose corn syrup consumption and excessive fructose consumption, and factors that harm human health, like obesity, ischemic heart disease, metabolic dysfunction, increased lipid profile levels, and hepatic insulin resistance [40]. Insulin resistance, liver lipid peroxidation, dyslipidemia and inflammatory cytokines have all been linked to fructose. Rats fed fructose-rich diets are widely accepted models for NAFLD and NASH [41].

In the positive control group of the current study, the induced NAFLD significantly increased liver enzyme levels (AST, ALT and ALP) that reflect and indicate hepatocyte damage [17], 34]. The biochemical changes in the liver of the NAFLD-induced rats (G2) caused a significant increase in serum levels of ALT, AST, ALP, albumin, TP, and bilirubin. Serum albumin levels were found to be lower in NAFLD-induced rats and associated with elevated liver enzymes, indicating that the synthetic function of the liver was affected due to liver injury [42]. In G3 treatment with flaxseed protected against liver injury by decreasing or normalization of the serum AST and ALT is ascribed to its content of 50–60 % omega-3 fatty acids in the form of linolenic acid [23], 24], 42], 43].

In the current study, the effect of a combination of flaxseed oil and probiotics on the liver. Briefly, co-administration of probiotics or flaxseed oil significantly improves liver functions and enzymes (ALT, ALP, AST, TP, albumin, bilirubin) in NAFLD rats, thus signifying the beneficial effect of probiotics and flaxseed oil on the NAFLD. Wong et al. [27] stated that probiotics improve liver enzymes in NAFLD patients. In addition, probiotics improved AST levels and insulin resistance. An Improvement in the liver enzymes and lipid profile was observed in this study by using a combination of both flaxseed oil and probiotics in G5 than using flaxseed oil or probiotics alone in G3 and G4, respectively. The current result is in the line that probiotic therapies decrease liver fat deposition, cholesterol, triglycerides, uric acid, and HOMA IR in animal and NAFLD patients [27], 44].

Probiotic therapies in both animals and humans reduced liver fat accumulation, decreased cholesterol, triglycerides and uric acid, and improved insulin resistance [45], 46]. A diet rich in omega-3 fatty acids improved the intra-hepatic triglyceride content and improved steatohepatitis in rats with fatty liver [34]. In this study, probiotics showed a beneficial role in NAFLD treatment. Probiotics may lower cholesterol by being incorporated into the cellular membrane and sequestering cholesterol from the gut [47], or by converting cholesterol to coprostanol, which is eliminated in the stool and potentially lowers cholesterol absorption in the intestine [22], 48]. Furthermore, omega-3 reduces endogenous lipid synthesis by blocking the expression and processing of genes that activate the transcription of various lipogenic and glycolytic genes in response to elevated glucose and insulin levels [34], 49].

The current study also showed that the oxidative stress parameters were increased in the NALD (G2) rats. In NAFLD an interaction between host genetics and environmental factors supports a key role in reactive oxygen species (ROS) generation that causes oxidative stress in the progression of NAFLD, which may be connected to oxidative stress in hepatocytes and peripheral insulin resistance [42]. The increased amounts of free fatty acids may cause “electron leakage” (due to the reduced activity of electron transport chain complexes), which leads to the generation of ROS and a loss in antioxidant defense [50]. In addition, sensitive lipase enhances lipolysis in adipose tissue, increasing free fatty acid levels while suppressing beta-oxidation and the formation of ROS [42]. In addition, the reduction in hepatic steatosis by treatment with flaxseed oil mixed with probiotics in G5 showed a decrease in oxidative stress markers and an increase in antioxidant markers compared to NAFLD induced group, suggesting that omega-3 accompanied by probiotics may play a protective role in preventing the progression of liver damage in NAFLD and could be useful as a prognostic marker of response to antioxidant treatment. This result is consistent with the confirmed role of probiotic administration in liver steatosis, liver enzymes, TNF-α and liver stiffness in patients with NAFLD [22], 51].

The protective effect of flaxseed oil in this study is consistent with previous investigations in reducing the development of pro-inflammatory markers such as IL-1β, TNF-α and IL-6 [52]. In addition, probiotics reduced TNF-α levels compared to the placebo group, which matches the current findings [53]. The novelty of the current study is in evaluating the protective effects of the combined omega-3 fatty acids in flaxseed oil with probiotics in treating NAFLD, and measuring inflammatory cytokines (TNF-α and IL-6 levels). The findings of the current study showed a significant increase in MDA concentration, TNF-α and IL-6 levels in NAFLD-induced rats, indicating a condition of hepatic inflammation. These inflammatory cytokines, particularly IL-6 and TNF-, play an important role in inflammation because TNF-α regulates cytokine production and the progression of the inflammatory response, whereas a rise in MDA oxidative marker concentration is associated with oxidative stress [54]. The flaxseed oil combined with probiotics reduced the pro-inflammatory processes and had positive effects on several components of the oxidative stress response [55]. On the other hand, the anti-inflammatory effects of omega-3 have been linked to the activation of free fatty acids receptors, which results in the preservation of inhibitors of nuclear factor [23], 24], 56], whereas probiotics exhibit an anti-apoptotic effect which is attributed to their ability to suppress cytokine-induced apoptosis [57]. The findings of the current investigation showed that omega-3 fatty acids of flaxseed oil accompanied by probiotics reduced augmented lipid peroxidation marker (MDA) and inflammation markers (IL-6, TGF-β and TNF-α), which confirms a positive correlation between measured oxidative stress and inflammatory markers [23], 24], 58].

In the current study, probiotics also lowered AST, ALT and TNF- α, which are all linked to the induction and consequences of NAFLD, according to a meta-analysis which is consistent with Yang et al. [59] and Firat et al. [60] who reported a positive correlation between liver injury marker and inflammatory cytokines. In addition, the current study showed the presence of positive correlations between estimated antioxidants (SOD, CAT, and TAC) and each other and negative correlations between lipid peroxidation biomarkers (MDA), and between the estimated antioxidants (SOD, CAT, and TAC) and the inflammatory cytokines (TNF-α, TGF-β, IL-6). This result is consistent with the studies confirming a correlation between omega-3 supplements as anti-inflammatory and antioxidant effects by lowering pro-inflammatory cytokines and reducing oxidative stress [23], 24], 61]. In addition, one of the most important cytokines accelerating hepatic fibrosis is the transforming growth factor TGF-β which influences many pathophysiological processes, like inflammation, tissue repair, cell migration and differentiation, and apoptosis [62].

The current study supports previous results that have demonstrated high levels of TGF-β in NAFLD-induced groups as a possible therapeutic target, *de novo* lipogenesis, bile acid-farnesoid X receptor axis, incretins and fibroblast growth factor-dependent pathways, inflammation and injury [63]. In the current study, the inhibitory effect of probiotics and omega-3 on TGF-β in NAFLD-induced rats is consistent with that of Cheng et al. [64] and Kelling et al. [65], respectively.

The present finding is in line with previous results that ROS can act as a stimulator of the TGF-β pathway, resulting in fibrosis development stated a positive correlation between oxidative stress and TGF-β, because ROS induces the TGF-β signaling pathway through many mechanisms, including matrix metalloproteinases activation, stimulation of TGF-β expression and Enhancement of TGF-β release via activation of the latency-associated peptide. It is also worth mentioning that, TGF-β stimulates the expression of NOX5 (NADPH Oxidase 5) in a ROS-dependent mechanism [23], 24], 66].

Also, this study indicated associations between inflammation in NAFLD-induced rats and inflammatory cytokines including TNF-α, L-6 and fibrosis with TGF-β, which confirm the positive correlation between inflammatory markers. Chen et al. [67] reported a significant increase in energy intake and body weights of NAFLD rats compared to the control group. Significant differences in the weights of the studied groups; the weight of rats in the flaxseed and the probiotics-treated group showed the nearest weight to the negative control group which is consistent with that of Kobyliak et al. [68].

The gross appearance of livers from the group supplemented with both flaxseed oil and probiotics had a similar finding as those of the negative control group. The hepatic tissue was yellow and greasy in NAFLD-induced rats, indicating fatty liver changes. In addition, there were statistically significant differences in the weights of the livers across groups. Histopathology of the liver of the induced NAFLD rats was verified and showed injured hepatic tissues, supporting previous studies [9], 41]. The NAFLD-induced rats in the positive control group (G2) showed macro and microvesicular steatosis in the histopathological images. In addition, the rats treated with flaxseed oil accompanied by probiotics (G5) effectively abrogated NAFLD-induced rats [2], 51], 68]. Rats fed on flaxseed oil accompanied by probiotics showed less steatosis than other treated groups, which improves the degree of steatosis after. Also, histopathological evaluation of the liver in rats using omega-3 accompanied by probiotics revealed less microvesicular steatosis than a single use of flaxseed oil or probiotics alone [9], 41]. The present study demonstrated more significant improvement in hepatic steatosis and hepatic lipid accumulation caused by the effects of omega-3 accompanied by probiotics compared to probiotics alone. These were in agreement with the result from Kobyliak et al. [23], 24], 68]. More studies are recommended on many rats for prolonged duration to confirm the therapeutic role of combined administration of food containing omega 3 and probiotics on hepatic steatosis. More studies are needed to clarify exactly the impact of which microbe of the 14 strains with the omega-3 fatty acids in reducing NAFLD.

## 5 Conclusions

The protective effects of flaxseed oil rich in omega-3 accompanied by probiotics on NAFLD-induced rats due to high-fructose corn syrup exposure are linked to restoring antioxidant status, inhibiting lipid peroxidation, and inflammation in the treated NAFLD rats. As a result, omega-3 of flaxseed oil and probiotics can function as nutraceuticals that are already available as prescriptive or non-prescriptive supplements and possible therapeutic therapy candidates for target organ injuries caused by oxide-inflammatory responses. It can be concluded that flaxseed oil and probiotics, separately and in combination, confer hepatoprotective effects against induced NAFLD in rats.

## Supplementary Material

Supplementary Material

Supplementary Material
