# Supplementary material for: Flaxseed oil and probiotics protect against induced nonalcoholic fatty liver disease (NAFLD) in male rats
Source: Open Life Sci. 2026 Feb 18;21(1):20251255. doi: 10.1515/biol-2025-1255 (PMC12917596; doi:10.1515/biol-2025-1255)
Supplement: Supplementary file 1 — Supplementary Material [file j_biol-2025-1255_suppl_001.docx]

**Supplementary table 1:** Plasma concentrations of liver enzymes (ALT, AST), albumin, total protein (TP), bilirubin, alkaline phosphatase (ALP) in the studied groups.

| **Variables** | **ALT (U/L)** | **AST (U/L)** | **ALP Mcg/L)** | **Total Protein**  **(g/dL)** | **Bilirubin**  **(mg/dL)** |
| --- | --- | --- | --- | --- | --- |
| **G1 (Negative control)** | 18.1 ± 3.28 | 17.8±2.8 | 157.0±3.9 | 6.6±.0.15# | 0.40±.0.21 |
| **G2 (positive NAFLD)** | 74.8±6.0* | 65.0±22.6* | 218.8±6.1* | 3.9±.0.17* | 1.9±.0.31* |
| **G3 (Flax seed oil)** | 44.1±2.3*# | 43.9±2.4*# | 186.4±2.4*# | 5.4±.0.12*# | 0.84±.0.32*# |
| **G4 (Probiotics)** | 57.9±3.1*# | 56.1±3.8*# | 196.8±4.4*# | 4.5±.0.14*# | 1.3±.0.15*# |
| **G5 (Flax seed oil + Probiotics(** | 32.3±2.2*# | 33.2±2.0*# | 173.5±3.5*# | 6.0±.0.11*# | 0.65±.0.26*# |

* indicates a statistically significant difference (P < 0.05). (*) Significant at p<0.05 compared to the negative control group (G1), (#) Significant at p< 0.05 compared to the positive NAFLD-induced control group (G2).
