# Supplementary material for: Flaxseed oil and probiotics protect against induced nonalcoholic fatty liver disease (NAFLD) in male rats
Source: Open Life Sci. 2026 Feb 18;21(1):20251255. doi: 10.1515/biol-2025-1255 (PMC12917596; doi:10.1515/biol-2025-1255)
Supplement: Supplementary file 2 — Supplementary Material [file j_biol-2025-1255_suppl_002.docx]

# Supplementary Table 2: Serum Concentrations of oxidative stress parameters and antioxidants biomarkers, in the studied groups.

| **Variables** | **TAC**  **mM/L** | **SOD**  **(U/L)** | **CAT**  **(U/L)** | **MDA**  **nM/ ml packed cells** |
| --- | --- | --- | --- | --- |
| **G1 (Negative control)** | 7.60±0.32 | 621.30±14.65 | 8.57±.88 | 1.42±0.54 |
| **G2 (positive NAFLD)** | 1.05±0.29^*^ | 260.60±.22.06^*^ | 1.19±0.28^*^ | 11.21±0.82^*^ |
| **G3 (Flax seed oil)** | 3.51±0.26^*#^ | 437.40±.23.42^*#^ | 4.07±0.29^*#^ | 5.98±0.53^*#^ |
| **G4 (Probiotics)** | 2.47±0.52^*#^ | 344.80±.17.42^*#^ | 2.64±0.38^*#^ | 7.84±0.46^*#^ |
| **G5 (Flax seed oil +**  **Probiotics(** | 5.85±0.29^*#^ | 493.60±16.71^*#^ | 6.00±0.39^*#^ | 3.46± 0.73^*#^ |

* significant at P < 0.05 compared to the negative control group (G1) and # significant at p< 0.05 as compared to control NAFLD-induced rats (G2).
